# Occlusal reconstruction of a patient with ameloblastoma ablation using alveolar distraction osteogenesis: a case report

**DOI:** 10.1186/s13005-020-00227-1

**Published:** 2020-06-02

**Authors:** Yoshihito Ishihara, Hikaru Arakawa, Akiyoshi Nishiyama, Hiroshi Kamioka

**Affiliations:** 1 Department of Orthodontics, Okayama University Graduate School of Medicine, Dentistry and Pharmaceutical Sciences, 2-5-1 Shikata-cho, Kita-ku, Okayama, 700-8525 Japan; 2 Department of Oral Rehabilitation and Regenerative Medicine, Okayama University Graduate School of Medicine, Dentistry and Pharmaceutical Sciences, Okayama, Japan; 3 Department of Oral and Maxillofacial Surgery, Okayama University Graduate School of Medicine, Dentistry, and Pharmaceutical Sciences, Okayama, Japan

**Keywords:** Ameloblastoma, Alveolar distraction osteogenesis, Implant anchorage, Postoperative malocclusion

## Abstract

**Background:**

Ameloblastoma is one of the most common benign odontogenic neoplasms. Its surgical excision has the potential to lead to postoperative malocclusion. In this case report, we describe the successful interdisciplinary orthodontic treatment of a patient with ameloblastoma who underwent marginal mandibulectomy.

**Case presentation:**

A woman of 20-year-old was diagnosed with ameloblastoma, and underwent marginal mandibulectomy when she was 8 years of age. She had an excessive overjet (11.5 mm) and a mild open bite (− 1.5 mm) with a severely resorbed atrophic edentulous ridge in the area around the mandibular left lateral incisor, canine and first premolar. An alveolar bone defect associated with tumor resection was regenerated by vertical distraction osteogenesis (DO). Subsequently, 3 dental implants were placed into the reconstructed mandible. Orthodontic treatment using implant-anchored mechanics provided a proper facial profile with significantly improved occlusal function. The occlusion appeared stable for a 7-year retention period.

**Conclusions:**

These results suggest that surgically assisted and implant anchored-orthodontic approaches might be effective for the correction of such malocclusions.

## Background

Ameloblastoma, which is one of the most common benign odontogenic neoplasms, generally presents in the jaw bone. It accounts for approximately 1% of oral tumors and cysts of the jaw. Approximately 80% of ameloblastomas occur in the mandible, mainly in the third molar region [1, 2]. With regard to the treatment of ameloblastoma, surgical excision with surrounding tissues is still the general approach since it clinically appears as an aggressive, asymptomatic, and slow growing tumor with high rate of recurrence [3, 4].

Patients with ameloblastoma who undergo surgical excision frequently show postoperative malocclusion because the teeth adjacent to the tumor should be extracted or are displaced by tumor growth. Additionally, the resorptive pattern of the jaw after the dentition has been lost, which often leads to a vertical alveolar discrepancy. An interdisciplinary approach for the reconstruction of the dentofacial region has been proposed to overcome the aesthetic and functional disabilities caused by surgical excision. Orthodontic treatment can provide an important role in creating an optimum occlusal relationship and sufficient space to allow for successful reconstruction of the affected region of the jaw in this interdisciplinary approach [5]. However, few reports have described such an interdisciplinary orthodontic treatment to optimally address the underlying dentofacial problems after the surgical removal of ameloblastomas in adolescents, since the tumor is considered a rarity in young people, who account for approximately 10–15% of all reported cases [6, 7].

We herein describe the successful orthodontic occlusal reconstruction of a patient with ameloblastoma ablation who demonstrated Class II malocclusion with an excessive overjet, open bite, and a severely resorbed atrophic edentulous ridge in the area around the mandibular left lateral incisor, canine and first premolar. This case report provides further evidence of the validity of orthodontic occlusal reconstruction by the concomitant use of implant anchorages, and implicates some functional interactions.

## Case presentation

### Diagnosis and etiology

**Fig. 1:**
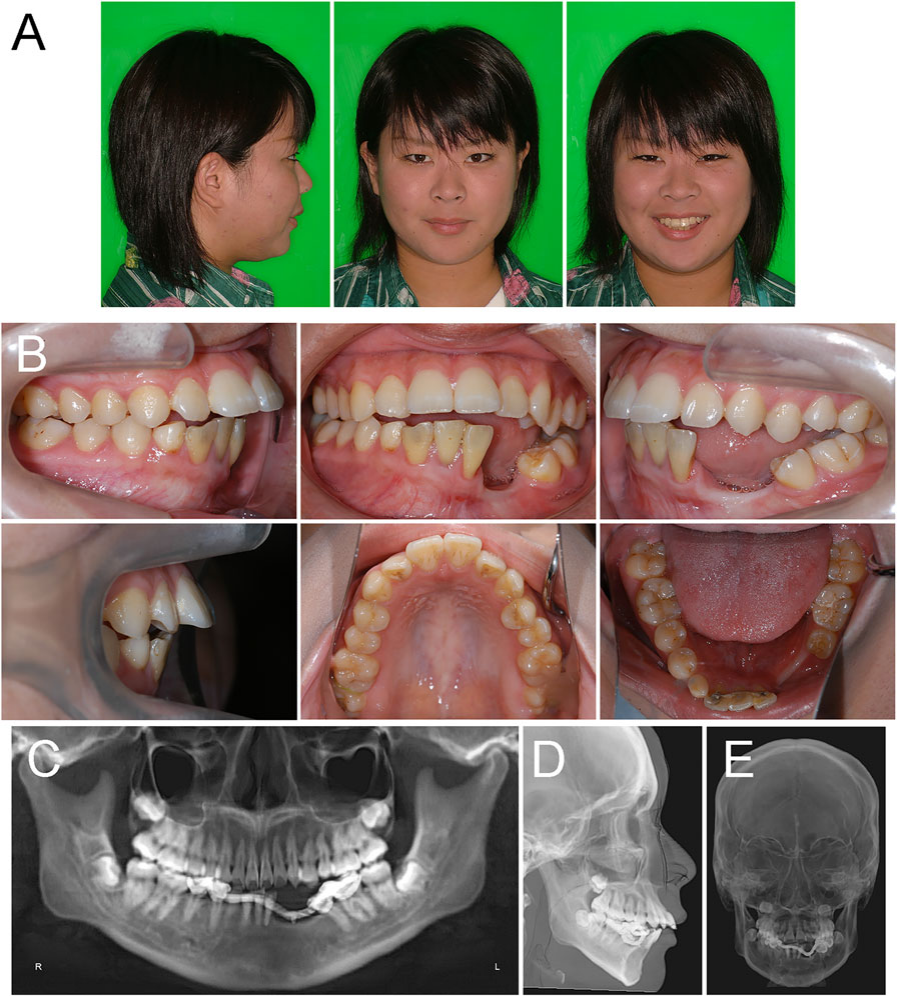
Pre-treatment recordings. **a** Extraoral photographs. **b** Intraoral photographs. **c** A panoramic radiograph. **d** A lateral cephalogram. **e** A posteroanterior cephalogram

A woman of 20 years and 2 months of age was referred to the Department of Orthodontics in Okayama University Hospital. She was diagnosed with ameloblastoma and underwent marginal mandibulectomy from the distal surface of the left central incisor to the left first premolar when she was 8 years of age. Her chief complaints were protruding maxillary incisors, functional disability, and aesthetic impairment due to surgical ablation. Extraoral examination showed that she had a symmetrical face, convex facial profile, an acute nasolabial angle, and protruded and incompetent lips (Fig. 1a). Intraoral examination revealed an excessive overjet of 11.5 mm combined with a mild open bite of − 1.5 mm. Angle Class I molar relationships were observed on both sides, while the incisor relationship was Class II. Although the maxillary dental midline almost coincided with the facial midline, the mandibular dental midline had deviated 2.0 mm toward the left of facial midline (Fig. 1b). Severe gingival recession was detected in the left lower central incisor. A dental panoramic tomogram confirmed root canal therapy on the lower incisors, and the absence of the lower left lateral incisor, canines, and first premolar, which was associated with ameloblastoma excision (Fig. 1c). The patient exhibited symptoms of temporomandibular disorder with a reciprocal clicking on the right side without pain. The interincisal distance on maximum mouth opening without pain was 49 mm. An occlusal-force recording system (Dental Prescale and Occluzer, Fuji Film, JAPAN) calculated that the occlusal force and occlusal contact area were 571 N and 13.4 mm^2^, respectively (Table 1).

**Table 1:**
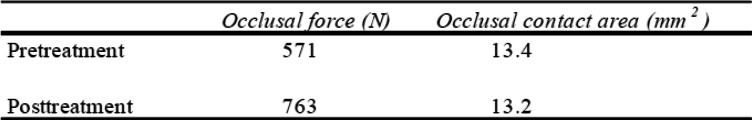
The changes in the occlusal function following orthodontic occlusal reconstruction

**Fig. 2:**
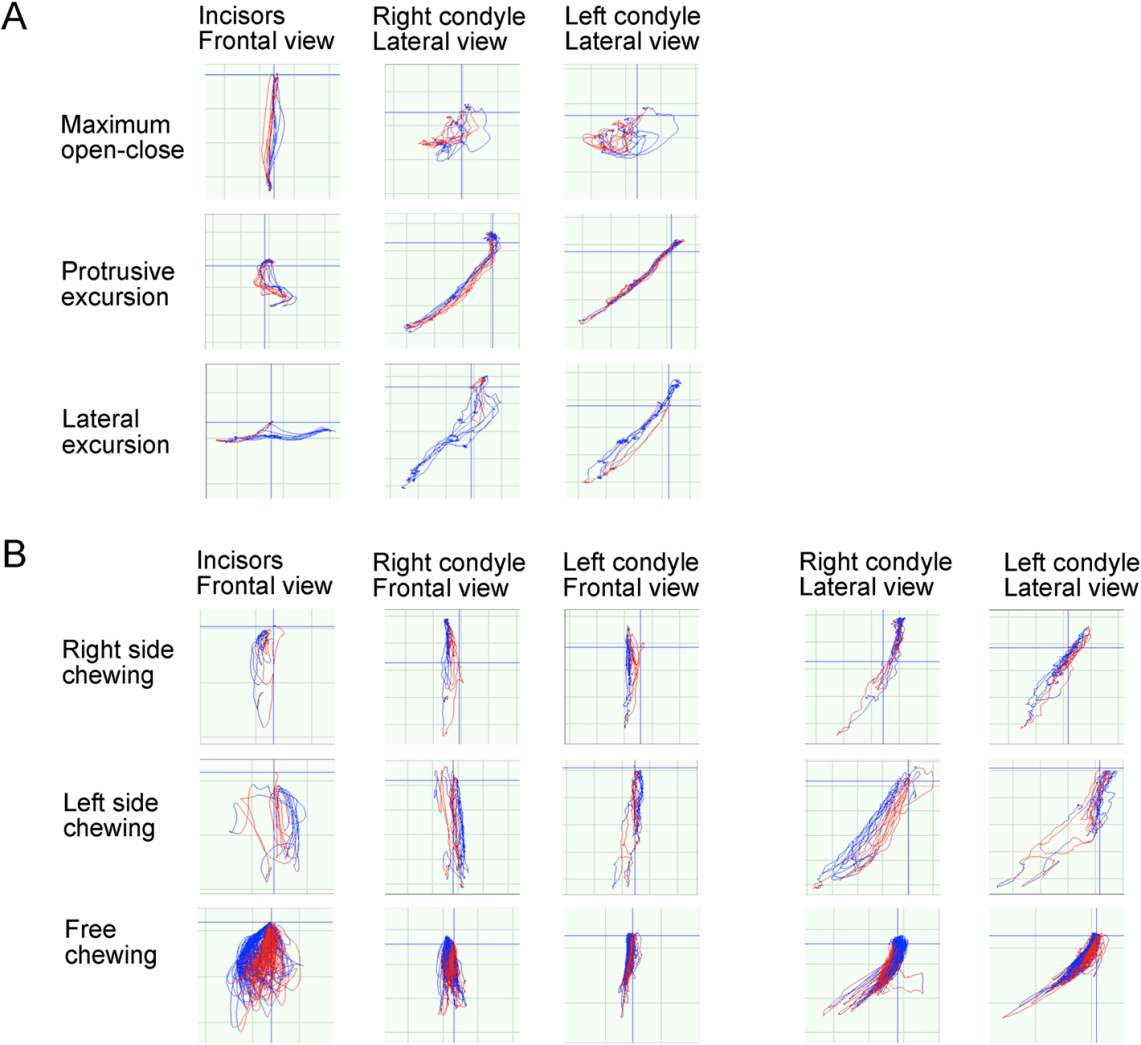
The pre-treatment condylar motion and incisal paths. **a** Maximum open-close and excursive movements. **b** Unilateral and free chewing movements. The colors in red and blue lines indicate the opening and the closing phase, respectively

The cephalometric examination of the patient indicated a skeletal Class II jaw-base relationship due to the relatively anterior position of the maxilla (ANB, 9.0°; SNA, 85.5°; SNB, 76.5°) with a steep mandibular plane angle (FMA, 36.0°) (Table 2) compared to the Japanese female norms [8]. The upper lip position was protruded against the aesthetic E-line (E-line to upper lip, + 3.5 mm), which was possibly associated with the labial inclination of the upper incisors (U1-FH, 127°: Table 2). An optoelectronic jaw tracking system with 6-degrees-of-freedom (Gnathohexagraph system, Ono Sokki Ltd., JAPAN) [9] showed the unstable motion of the mandible and both sides of the condylar heads during maximum opening and closing, protrusive excursion, or lateral excursion of the jaw movements (Fig. 2a). Unstable patterns of jaw movement were noted in a chewing test (Fig. 2b).

**Table 2:**
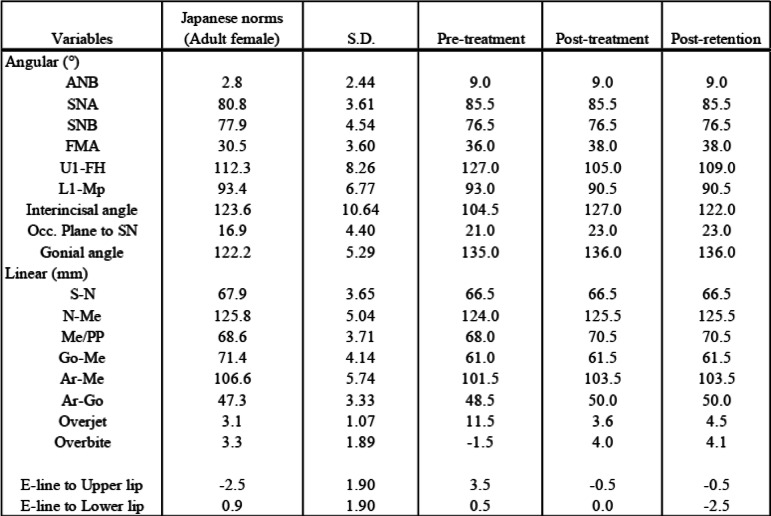
A summary of the cephalometric findings

*Occlusal force (N) Occlusal contact area (mm 2)*

Pretreatment 571 13.4

Posttreatment 763 13.2

### Treatment objectives

**Fig. 3:**
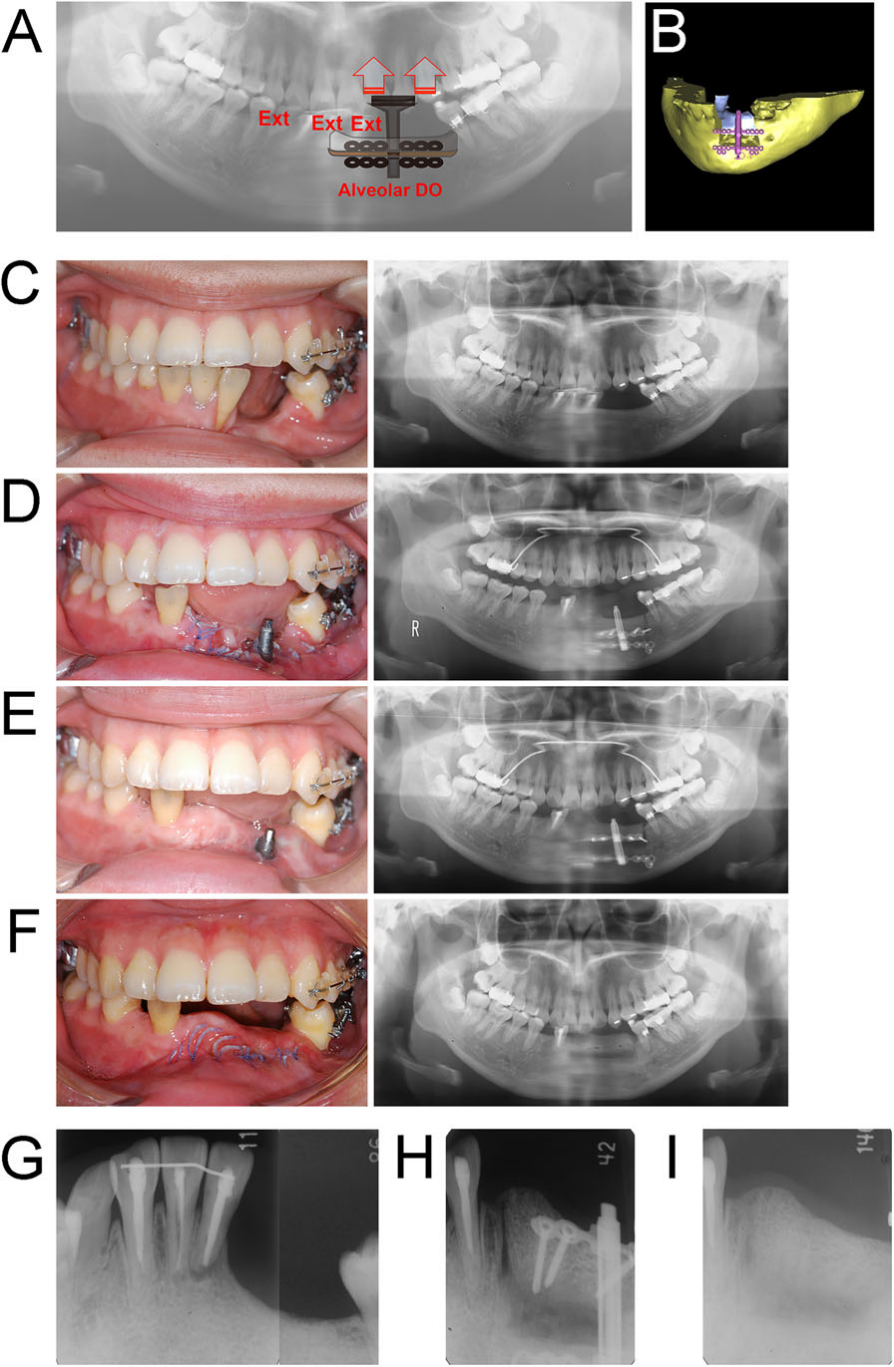
The treatment progress during alveolar distraction osteogenesis (DO). **a** A schematic illustration of the alveolar DO. **b** Three-dimensional prediction of the postoperative outcome with computed tomography. **c** Before alveolar DO. **d** After surgery for alveolar DO. **e** After the completion of alveolar DO. **f** After the surgical removal of instruments for alveolar DO. **g**-**i** Representative dental radiographs showing gradual changes in the alveolar bone during treatment. **g** Pre-treatment (**h**) After the completion of the alveolar DO. **i** After the surgical removal of the instruments for alveolar DO

The patient was diagnosed with a skeletal Class II jaw-base relationship due to the anteriorly positioned maxilla, protruding upper incisors with an open bite, and vertical defects of the edentulous ridge on the mandible caused by the marginal mandibulectomy for ameloblastoma. Alveolar distraction osteogenesis (DO) was planned for the atrophic edentulous ridge in order to obtain adequate vertical bone height and allow the optimal placement of a dental implant with bone augmentation as the first step in the overall treatment (Fig. 3a, b). The second step of treatment aimed to camouflage the anteroposterior skeletal discrepancy, improve facial esthetics, create functional and aesthetic occlusion, and correct an excessive overjet with mild anterior open bite by retracting the upper incisors. In this case, the extraction of upper first premolars were effective to achieve the treatment objectives. Using skeletal anchorage could be considered to achieve maximum anchorage reinforcement.

### Treatment alternatives

The limited height of the dentate mandible could be a particular problem, especially when rehabilitation with dental implants is contemplated. Alternative treatmentfor vertical defects of the edentulous ridge include the use of bone grafting. The treatment of choice was previously considered the gold standard, however, their use required not only second-site surgery with possible donor site morbidity, but also the additional oral dysfunction. This method also has the disadvantages of undergoing resorption and contraction of adjacent soft tissues, particularly during the initial 6 months [10]. Furthermore, there is a higher risk of surgical site infection in comparison to alveolar DO [11]. A conservative prosthodontic option does not allow the insertion of regular length prosthesis in the patients with atrophic mandibular ridge. Insufficient vertical height of the mandible also leads to overloading of osseointegrated implants, which may influence the long-term prognosis of the prosthetic restoration. She and her family decided to undergo vertical DO with interdisciplinary orthodontic treatment after a thorough discussion.

### Treatment progress

Before the osteodistraction procedure, the patient’s lower central incisors and the lower right deciduous canine were extracted because of the extensive periodontal damage and poor prognosis (Fig. 3c-d). Alveolar DO was performed in the vertical direction using a bone plate type distractor (Track-Plus; KLS/Martin, Jacksonville, FL, USA) corresponding to the expected distraction distance of 12 mm over 4 weeks, and the consolidation period was 16 weeks. After alveolar DO, intraoral photographs showed simultaneous osteogenesis and neohistogenesis (Fig. 3e). The distraction devices were removed under general anesthesia (Fig. 3f). Newly formed bone was confirmed by panoramic radiograph and dental radiographs (Fig. 3g-i).

**Fig. 4:**
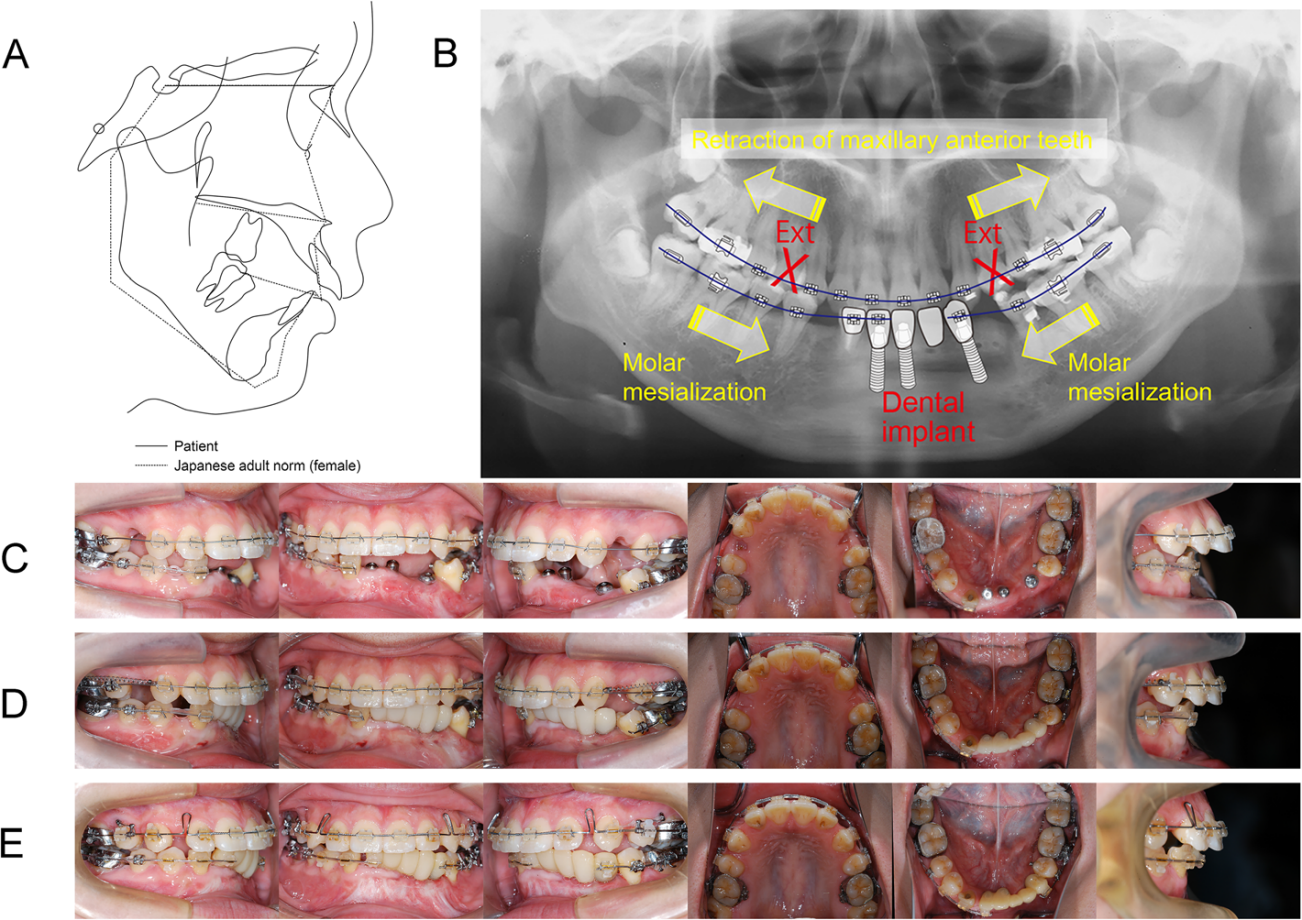
The treatment progress during comprehensive orthodontic treatment. **a** The pre-treatment cephalometric tracing superimposed on an average profilogram **b** A schematic illustration of the comprehensive orthodontic treatment. **c** At the initiation of leveling. **d** At the initiation of simultaneous mandibular molar mesialization and maxillary canine retraction. **e** At the start of maxillary incisors retraction

The second step of orthodontic treatment was initiated to correct the antero-posterior discrepancies by retracting the upper anterior incisors when the patient was 23 years and 1 month of age (Fig. 4a). Figure 4b is a schematic illustration showing the second step of the orthodontic treatment plan. Prior to start the second step of orthodontic treatment, 3 dental implants were placed into the reconstructed mandible. Following the extraction of the maxillary first premolars, a 0.018-in. slot pre-adjusted edgewise appliance with a 0.016-in. nickel-titanium (Ni-Ti) wire was placed in the both arches to initiate leveling (Fig. 4c). Three months later, the upper and lower wires were changed to a 0.016 × 0.022-in. Ni-Ti arch wire. Five months after leveling and alignment, the abutments were connected with the dental implants, and the provisional prosthesis was cemented on the implant abutments utilized as an absolute anchorage to protract the lower molars. Simultaneously, a 0.016-in. stainless steel (SS) archwire was placed to retract the upper canines using 100-g Ni-Ti closed coil springs (Fig. 4d). The mesialization of the lower first molars were initiated after achieving the leveling and alignment of the mandibular arch. Two miniscrews (Absoanchor®; Dentos Ltd., Daegu, Korea) were inserted with the distal alveolus of the maxillary first molars to provide anchorage reinforcement. After the completion of upper canine retraction, a 0.016 × 0.022-in. SS archwire was installed to retract the upper incisors and complete the remaining space closure using closing-loop mechanics (Fig. 4e). Finishing and detailing was conducted with 0.017 × 0.025-in. SS wires in both arches. The total period of the second step of treatment was 56 months. Appliances were removed, and a lingual fixed retainer combined with a removable retainer was delivered in the upper arch.

### Treatment results

**Fig. 5:**
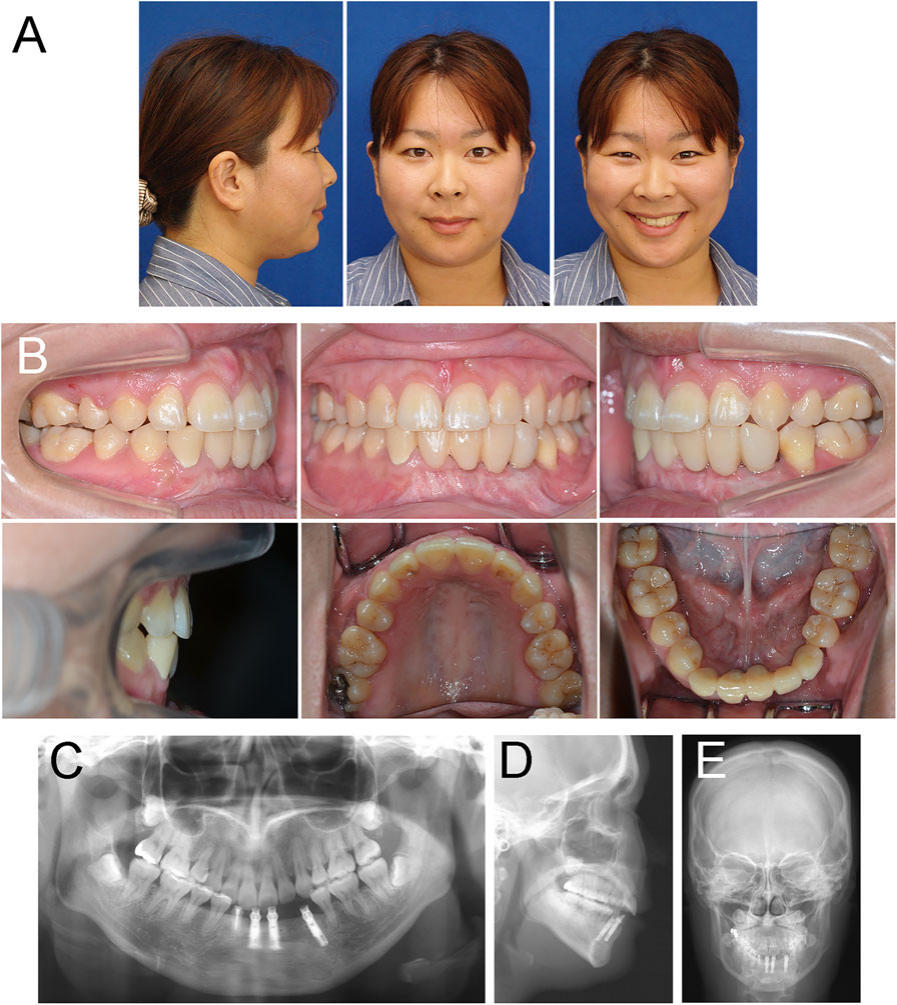
The post-treatment recordings. **a** Extraoral photographs. **b** Intraoral photographs. **d** A panoramic radiograph. **c** A lateral cephalogram. **e** A posteroanterior cephalogram

**Fig. 6:**
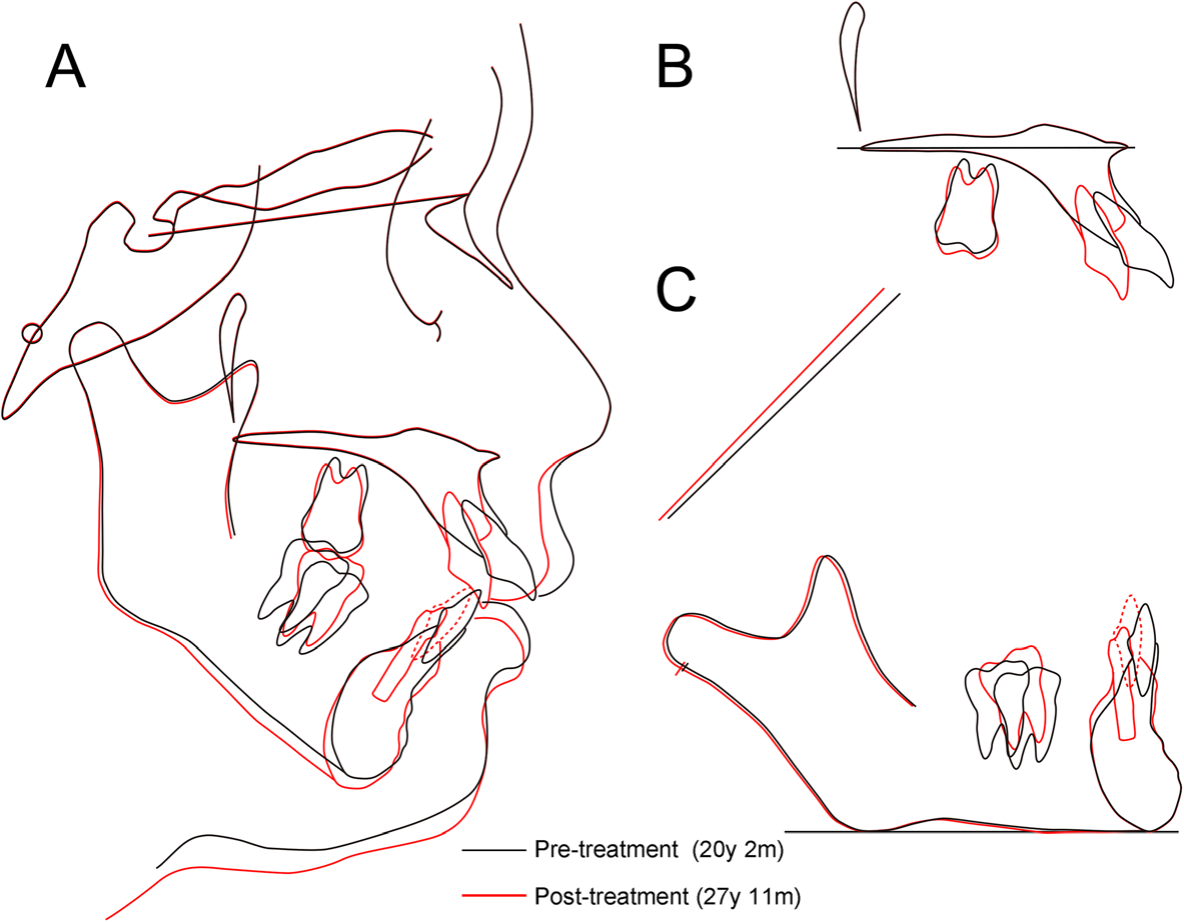
The superimposed cephalometric tracings at pre-treatment (*black*) and post-treatment (*red*). **a** The overall superimposition on sella-nasion plane at the sella. **b** The superimposition on palatal plane at the ANS. **c** The superimposition on mandibular plane at the menton

The comprehensive orthodontic treatment improved the facial profile with a favorable change in the lip posture and balance (Fig. 5a). Class I molar and canine relationships were obtained on both sides with normal overjet and overbite. The post-treatment intraoral photographs also showed well-aligned dentition (Fig. 5b). A panoramic radiograph indicated acceptable root paralleling and no obvious apical root resorption, except slight root resorption in the upper central incisors (Fig. 5c). Post-treatment cephalometric radiographs and superimpositions showed no marked skeletal changes (Figs. 5 d-e, and 6). The reduction of the excessive overjet was achieved by the lingual inclination of the upper incisors to camouflage skeletal Class II jaw-base relationship (Table 2).

**Fig. 7:**
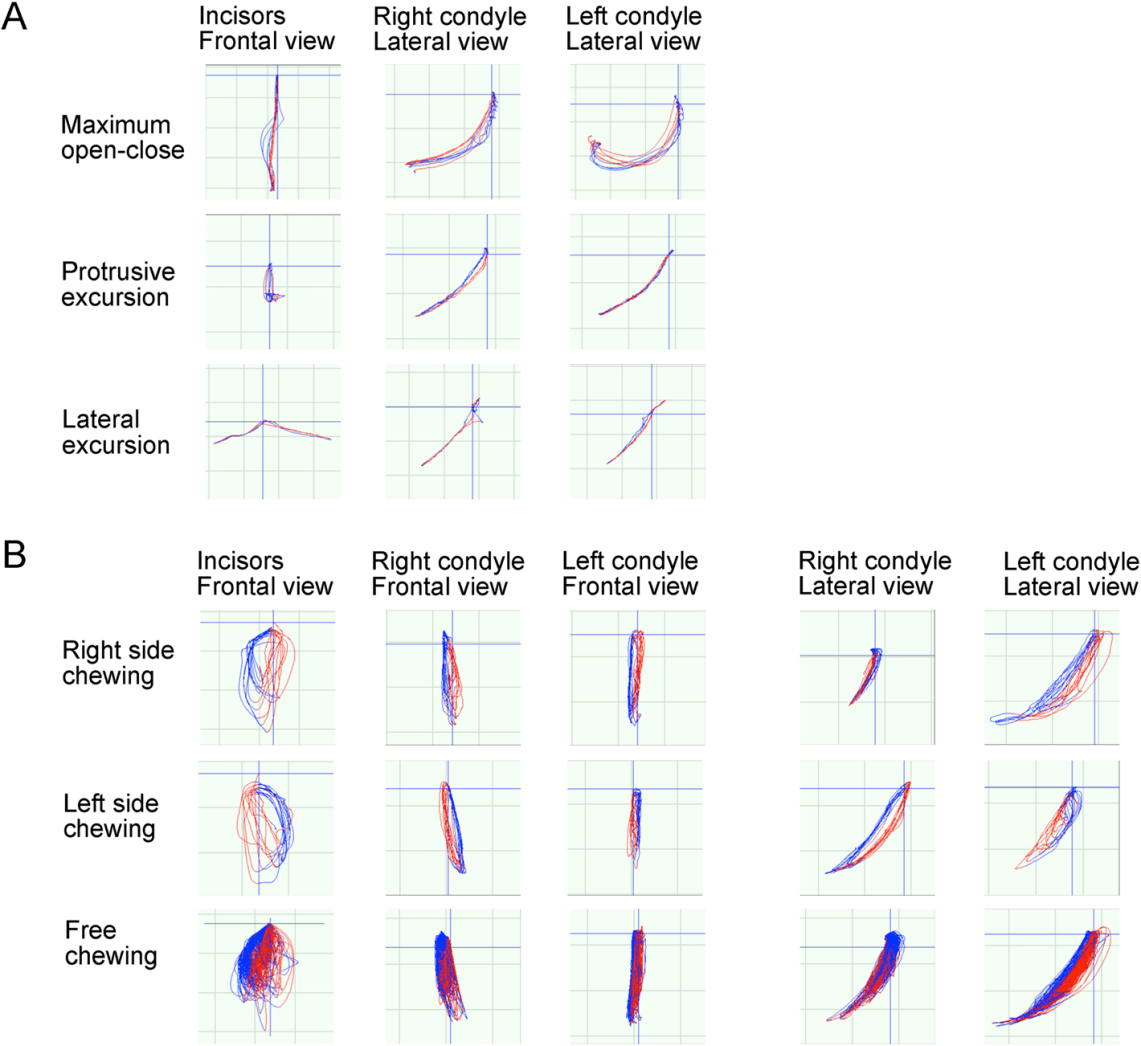
The post-treatment condylar motion and incisal paths. **a** Maximum open-close and excursive movements. **b** Unilateral and free chewing movements. The colors in red and blue lines indicate the opening and the closing phase, respectively

The pattern of the jaw movement revealed that the motion of the condylar head on both sides was stable with a good locus in the maximum open-close and excursive motions (Fig. 7a). A unilateral and free chewing gum test showed more stabilized motion of both condyles and lower incisors (Fig. 7b). The occlusal force slightly increased after the post-treatment period (Table 1).

**Fig. 8:**
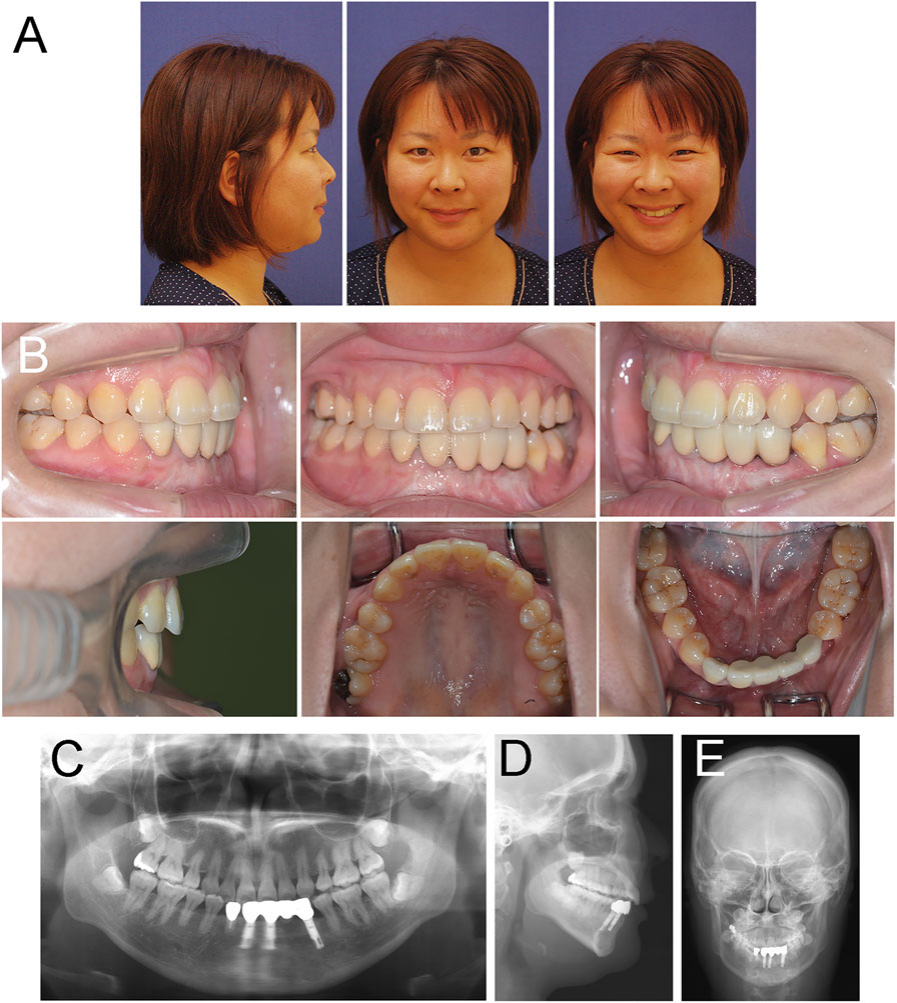
Two-year retention recordings. **a** Extraoral photographs. **b** Intraoral photographs. **c** A panoramic radiograph. **d** A lateral cephalogram. **e** A posteroanterior cephalogram

**Fig. 9:**
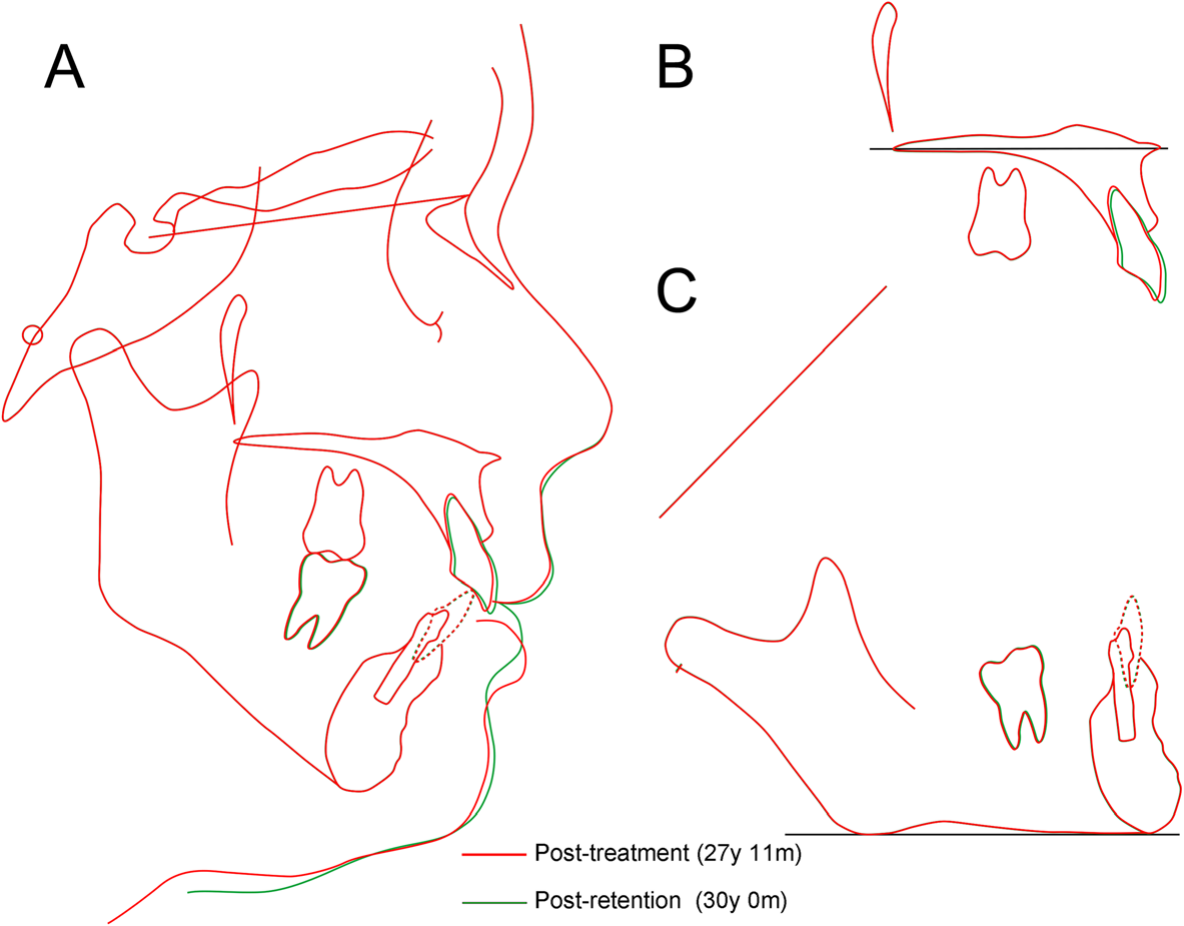
The superimposed cephalometric tracings at post-treatment (*red*) and two-year retention period (*green*). **a** The overall superimposition on sella-nasion plane at the sella. **b** The superimposition on palatal plane at the ANS. **c** The superimposition on mandibular plane at the menton

**Fig. 10:**
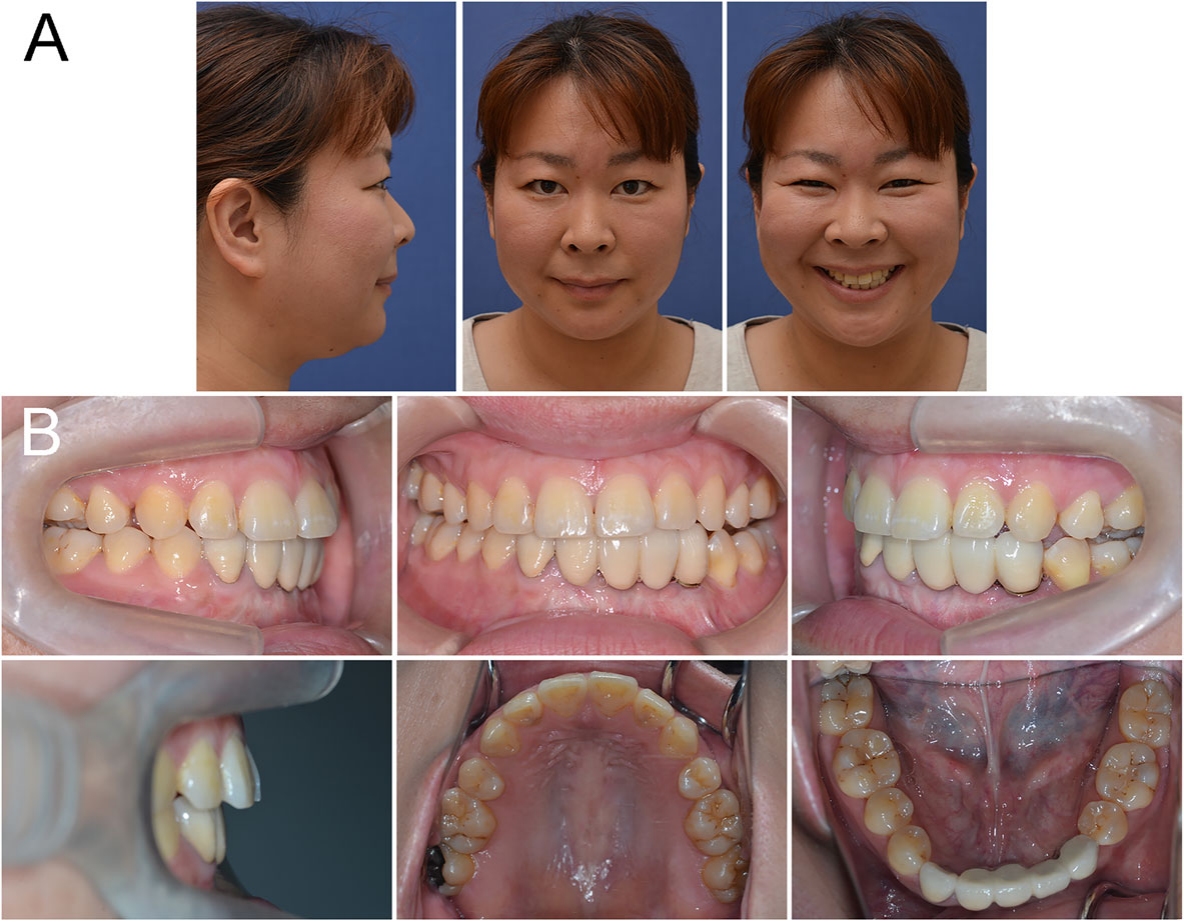
Seven-year retention recordings. **a** Extraoral photographs. **b** Intraoral photographs

The improved facial profiles achieved by the interdisciplinary orthodontic treatment were maintained after 24 months of retention. Intraoral photographs also showed that the acceptable occlusion with adequate overbite and overjet had been maintained with the exception the upper left molars (Fig. 8). Peri-implant soft tissues showed a healthy status. A post-retention cephalometric evaluation and superimposed cephalometric tracing showed no marked skeletal changes (Fig. 9). The overjet was slightly increased due to proclination of the upper incisors (Table 2). Follow-up at 7-years of retention confirmed that the patient’s occlusion was maintained. At present, she is still being followed (Fig. 10).

## Discussion

Alveolar DO is an efficient method for correcting alveolar deformities in ridge height and width [12, 13]. The principle of DO was established in the 1950s by the studies of Ilizarov, who showed that osteogenesis can be induced if bone is distracted along its long axis [14]. After corticotomy of the mandibular alveolar and rigid fixation with external devices, a callus develops between bone segments during slow activation at the distraction gap, and the newly formed callus matures to bone by fixation. This technique provides high-quality newly formed bone with improved bone dimensions in the vertical or horizontal aspects. We showed the orthodontic treatment of a patient with an atrophic edentulous ridge due to the surgical resection of an ameloblastoma. To our knowledge, the present study represents the first report of interdisciplinary orthodontic treatment including alveolar DO, which achieved long-term stability and an assessment of the stomatognathic function.

Implant-supported oral rehabilitation in atrophic edentulous areas has always been a challenge, particularly in the mandible, due to an insufficient bone volume to place implants of adequate dimensions and the presence of the inferior alveolar canal. Orthodontic tooth movement into the edentulous area is a possible option for enhancing the buccolingual ridge dimensions to serve as an implant site [15, 16]. We initially used an implanted prosthesis as an orthodontic anchorage to accomplish absolute mesial movement of the lower molars for both an adequate occlusal relationship and a progressive increase in bone volume, and secondarily as an abutment for a fixed prosthesis for maintaining the new-formed bone. It appears that the subsequent bone remodeling by orthodontic tooth movement also provided favorable dental implant therapy at the resorbed atrophic edentulous ridge at which marginal mandibulectomy had been performed. A previous report demonstrated the positive periodontal and functional findings in teeth that were orthodontically moved into edentulous areas [17]. However, in this procedure lateral root resorption should be considered as an inevitable side-effect, whereas apical root resorption is less common [18].

A favorable change in the facial balance with functional occlusion was achieved following the combined orthodontic and prosthodontic treatments. Such improvements might be explained by the correction of the excessive overjet and open bite for creating more stable jaw movement. In the present case, we used a miniscrew in the upper arch because maximum anchorage was required to retract the upper anterior teeth sufficiently. However, the superimposed cephalogram tracings at pre-treatment/post-treatment illustrated the extrusion of the upper first molars, which was possibly associated with the clockwise rotation of the mandible. Vertical control of the maxillary molars with miniscrew-aided mechanics might have been considered to be the more appropriate treatment choice [19]. This method would have led to the autorotation of the mandible in the counterclockwise direction, thereby increasing the overbite and improving the convex profile. The increased duration of second phase orthodontic treatment was affected by the cessation of treatment due to her hospitalization for pregnancy and miscarriage. The associated difficulties in physical and mental health probably added 1 year to the treatment time.

Post-treatment stability is another concern after the correction of excessive overjet and an open bite. With the exception of the buccally inclined maxillary second molars, favorable results were maintained after the 7-year postretention. On the other hand, further observation is also required because of uncertainty about the long-term prognosis of the area where the ameloblastoma was removed. Past reports have indicated that long-term follow-up is important, especially in the conservative treatment of unicystic ameloblastoma, due to the high rate of recurrence after tumor removal [20]. Although our patient underwent surgical resection, which was the preferred option for the management of recurrence [21], routine follow-up will be needed for a long time as part of the interdisciplinary approach.

## Conclusions

Our findings in this case report suggest that surgically assisted- and implant anchored-orthodontic treatment might be effective for occlusal reconstruction in a patient with a severely resorbed atrophic edentulous ridge. In addition, such an interdisciplinary approach has the potential to ensure the substantially improving the health and quality of life of patients with ameloblastoma ablation.

## Data Availability

Not applicable.

## Abbreviations

DO: Distraction osteogenesis
Ni-Ti: Nickel-titanium
SS: Stainless steel
